# Hospital Admission following Induced Abortion in Eastern Highlands Province, Papua New Guinea – A Descriptive Study

**DOI:** 10.1371/journal.pone.0110791

**Published:** 2014-10-17

**Authors:** Lisa M. Vallely, Primrose Homiehombo, Angela Kelly-Hanku, Antonia Kumbia, Glen D. L. Mola, Andrea Whittaker

**Affiliations:** 1 Sexual and Reproductive Health Unit, Papua New Guinea Institute of Medical Research, Alotau, Milne Bay Province, Papua New Guinea; 2 Sexual and Reproductive Health Unit, Papua New Guinea Institute of Medical Research, Goroka, Eastern Highlands Province, Papua New Guinea; 3 International HIV Research Group, School of Public Health and Community Medicine, University of New South Wales, Sydney, Australia; 4 Division of Obstetrics and Gynaecology, Eastern Highlands Provincial Hospital, Goroka, Eastern Highlands Province, Papua New Guinea; 5 School of Medicine and Health Sciences, University of Papua New Guinea, Port Moresby, Papua New Guinea; 6 School of Social Sciences, Monash University, Victoria, Australia; Indiana University, United States of America

## Abstract

**Background:**

In Papua New Guinea abortion is restricted under the Criminal Code Act. While safe abortions should available in certain situations, frequently they are not available to the majority of women. Sepsis from unsafe abortion is a leading cause of maternal mortality. Our findings form part of a wider, mixed methods study designed to identify complications requiring hospital treatment for post abortion care and to explore the circumstances surrounding unsafe abortion.

**Methods:**

Through a six month prospective study we identified all women presenting to the Eastern Highlands Provincial Hospital following spontaneous and induced abortions. We undertook semi-structured interviews with women and reviewed individual case notes, extracting demographic and clinical information.

**Findings:**

Case notes were reviewed for 56% (67/119) of women presenting for post abortion care. At least 24% (28/119) of these admissions were due to induced abortion. Women presenting following induced abortions were significantly more likely to be younger, single, in education at the time of the abortion and report that the baby was unplanned and unwanted, compared to those reporting spontaneous abortion. Obtained illegally, misoprostol was the method most frequently used to end the pregnancy. Physical and mechanical means and traditional herbs were also widely reported.

**Conclusion:**

In a country with a low contraceptive prevalence rate and high unmet need for family planning, all reproductive age women need access to contraceptive information and services to avoid, postpone or space pregnancies. In the absence of this, women are resorting to unsafe means to end an unwanted pregnancy, putting their lives at risk and putting an increased strain on an already struggling health system. Women in this setting need access to safe, effective means of abortion.

## Introduction

Of the 44 million abortions that took place globally in 2008 nearly half were considered unsafe [1]. An important distinction between a safe and an unsafe abortion is that a safe induced abortion has few health consequences for the woman whereas an unsafe induced abortion can pose a significant health threat to them, in terms of both morbidity and mortality [2], [3]. Forty per cent of women seeking induced abortion live in countries where it is legally restricted. But even where induced abortion is legal, access to such services is often poor [4]. The majority of unsafe abortions that take place every year occur in developing countries [5], frequently undertaken by individuals without the necessary skills to perform the procedure; alternatively they may be self-induced. The circumstances and environment in which unsafe abortion is performed may be aggravated by unhygienic conditions and interventions or incorrect administration of medication [5].

Unsafe, induced abortion procedures may involve the ingestion of harmful substances and physical means such as insertion of a foreign object or substance through the cervix and into the uterus, or external force, such as squeezing the abdomen [5]–[7]. More recently, it is suggested that the increasing availability and use of the E1 prostaglandin analogue, misoprostol, is replacing many of these risky methods of unsafe abortion [6], [8], [9]. It has been suggested that in developing countries, even when used incorrectly, severe complications and maternal deaths are lower with the use of misoprostol when compared to physical means of unsafe abortion [10], [11].

Over the past few decades there has been a decline in maternal mortality [12] however, mortality from unsafe induced abortion has remained the same, accounting for approximately 15% of all maternal deaths [12]. The majority of maternal deaths attributable to unsafe abortion occur in developing countries, frequently linked to lack of access to care during an emergency [5]. In addition to maternal deaths many more women suffer both short and long term health consequences [1], including haemorrhage, sepsis and infertility [6].

Whether legal or not, induced abortion is a sensitive topic and induced abortion is frequently stigmatized [1]. While this stigma may be perceived, or experienced, for those seeking both abortion and post abortion care, stigma is also reported in relation to service delivery and at the policy level [13], [14]. In countries where induced abortion is restricted or inaccessible due, for example, to legal socio-cultural or geographical barriers, seeking information on incidence, practices and outcomes is difficult. When abortion occurs in clandestine situations, it may not be reported or may be declared as a spontaneous abortion [1]. Using estimates of indirect methods, for example information relating to complications treated in hospital, studies on conditions of unsafe abortions and women’s reports through health and population surveys can provide some information [1]. However, these data need to be reviewed with caution and it can be reasonable to assume that they are an underestimate. Women living in rural areas with poor access to hospitals, which tend to be situated in urban areas, are unlikely or unable to obtain care if they have an abortion complication [4]. In addition, many women may have unsafe abortions, but no complications and therefore not present for hospital level care and having no detectable encounter with the health system. Maternal deaths may also occur in the community setting and remain unrecognised or unreported as an abortion related death.

As well as the health consequences for the individual woman, consequences of unsafe abortion result in increased costs to health systems: use of hospital beds, blood supplies, medication, operating theatres, anaesthesia and medical specialists all stretch a resource poor situation [4], [15]. Efforts to reduce unsafe abortion and to understand why and under what circumstances women resort to such abortions are crucial if maternal mortality due to unsafe abortion is to be reduced [16].

Papua New Guinea (PNG) has the highest maternal mortality ratio in the Oceania region and one of the highest in the world, with an estimated 594 maternal deaths per 100,000 live-births [12]. Sepsis due to unsafe abortion is reported as a leading cause of maternal mortality in PNG [17], [18]. The country has a low contraceptive prevalence rate for modern methods of family planning among married women (24%) and a high unmet need for family planning (27%) [19]–[21].

In PNG the legal framework surrounding abortion is contained within the Criminal Code Act of 1974 [22], which reproduces the 1899 Criminal Code Act of Queensland, adopted as the law of the colony of British New Guinea. Since 1993, following a request from the PNG Department of Health to the State Solicitor, an induced abortion may be undertaken to save a woman’s life and/or to preserve the woman’s physical and mental health, and can be legally performed by trained providers in safe conditions, provided there is agreement by two medical officers. However, in reality safe, induced abortions are not available for the majority of women in PNG and induced abortion is not available in any public health facility in PNG [17]. Although in every urban setting a health practitioner (usually a doctor in private practice) is willing to safely induce abortion, fees at private clinics are too expensive for the majority of Papua New Guineans [17].

As with many aspects of maternal health in PNG, there is a paucity of research relating to unsafe abortion. Work undertaken in the early 1990s and in 2011 relating to sexual and reproductive health indicates that women do seek and undertake illegal, unsafe, often self-induced abortion to end an unwanted pregnancy [23], [24]. To date no study has described women’s experiences, including seeking care post abortion, clinical presentation and management at hospital.

The findings presented in this paper form part of a wider, mixed methods study. The overall aim of the study was to identify the types of complications that require hospital treatment as a result of spontaneous and induced abortion and to explore the reasons why and under what circumstances women resort to induced abortion. Through a qualitative component, we explored the reasons why women resort to unsafe abortion, the techniques used, the events leading to hospital admission and the decision making processes relating to both the abortion and seeking post abortion care. The qualitative data relating to the wider study is presented elsewhere [25], [26]. In this paper we present demographic and clinical information for women presenting for hospital level care as a result of self-reported spontaneous and induced abortion. We describe the socio-cultural differences, clinical presentation and outcomes between the two groups of women. Timing and methods used for unsafe abortion are also described.

## Methods

### Ethics statement

This research was approved by the Medical Research Advisory Committee (MRAC 11.32) PNG in December 2011; the Institutional Review Board of the Papua New Guinea Institute of Medical Research (IRB 1201) in February 2012; and the University of Queensland Human Research Ethics Committee in Australia (LV080312) in March 2012. An amendment to protocol was approved in July 2012. Written consent was obtained from all those who participated in the semi structured interviews and case note review. To ensure anonymity all participants were assigned a unique study identity number.

### Study site

Situated in the Highlands region of PNG, the Eastern Highlands Province has an estimated population of 540,000; the majority live in rural areas. The Eastern Highlands Provincial Hospital in Goroka is the referral hospital for the province. In 2012 there were a total of 1,186 gynaecology admissions at the hospital and an estimated 15 births a day. In 2009, a retrospective study undertaken at the hospital identified puerperal sepsis and abortion related sepsis as leading causes of maternal mortality [17]. At the same hospital a prospective study, undertaken in 2011, identified misoprostol as the most widely used means to induce an abortion [27].

### Data collection

A prospective, mixed methods study of women presenting to the Eastern Highlands Provincial Hospital with complications following self-reported spontaneous and induced abortion was undertaken over a six month period. We sought to identify all women admitted to the hospital with suspected or confirmed abortion, including both spontaneous and induced abortion. All data collection was undertaken by one trained and experienced research midwife from the Papua New Guinea Institute of Medical Research (PNGIMR) and overseen by the principle investigator for the study.

Between 28^th^ May and 30^th^ November 2012, daily review of available admission records, including ward admission books, was undertaken by the research midwife at the emergency department, out-patient department, well woman clinic and the obstetric and gynaecology ward. We sought to identify any woman admitted with a complication indicative that an abortion may have taken place, including spontaneous abortion, safe abortion and unsafe abortion. Inclusion criteria were women identified in the admission book or through clinical records as bleeding during pregnancy; reporting an induced, illegal abortion; reporting a spontaneous abortion; excessive vaginal bleeding; lower abdominal pain with vaginal discharge/bleeding; fever with vaginal bleeding/discharge; foreign body in uteri or pelvic injury. In line with the PNG National Department of Health guidelines, abortion was defined as vaginal bleeding before 20 weeks gestation or fetal weight of less than 500 grams.

### Semi structured interviews

Semi structured interviews were introduced during the second month of data collection (27^th^ July 2012), following an amendment to the original research protocol and ethics approval. Semi structured interviews were included to ensure all cases of unsafe abortion, not revealed as such at the time of admission or not identified on clinical examination, were identified. Following informed consent procedures, a semi structured interview guide (Figure S1) was used to conduct interviews with women meeting the inclusion criteria. We sought to identify women’s reasons for seeking hospital level care, their reaction to the pregnancy and their feelings in relation to the pregnancy loss.

### Case note review

Following identification of women through the admission book, or following the semi structured interview, women identified reporting as either a spontaneous or induced abortion were approached and informed consent procedures completed. For women providing consent a piloted study specific case note record form was used to identify basic socio-demographic information, reproductive and obstetric history, presenting complaints, time between onset of symptoms and seeking care, diagnosis and clinical management received.

### Data management

All clinical data were entered into a study specific MS Access database by one member of the sexual and reproductive health team at the PNGIMR and was cleaned prior to analysis. All semi structured interviews were transcribed and translated by the research midwife. Transcripts were managed using NVivo 9 (QSR International Pty Ltd 2010), a qualitative data software programme.

### Data analysis

Statistical analyses were conducted in Stata 12.1 (StataCorp LP, Texas, USA) to compare socio-demographic, behavioural and clinical outcomes among women reporting spontaneous and induced abortion. Due to the modest sample size of the study, it was not possible to conduct multivariate analysis to examine independent risk factors. Semi structured interviews were reviewed and discussed between the principle investigator and the research midwife to identify additional cases of induced abortion not identified through the hospital admission records. Transcripts were reviewed to identify information relating to induced abortions, including gestation at abortion and abortion method used.

## Results

**Figure 1:**
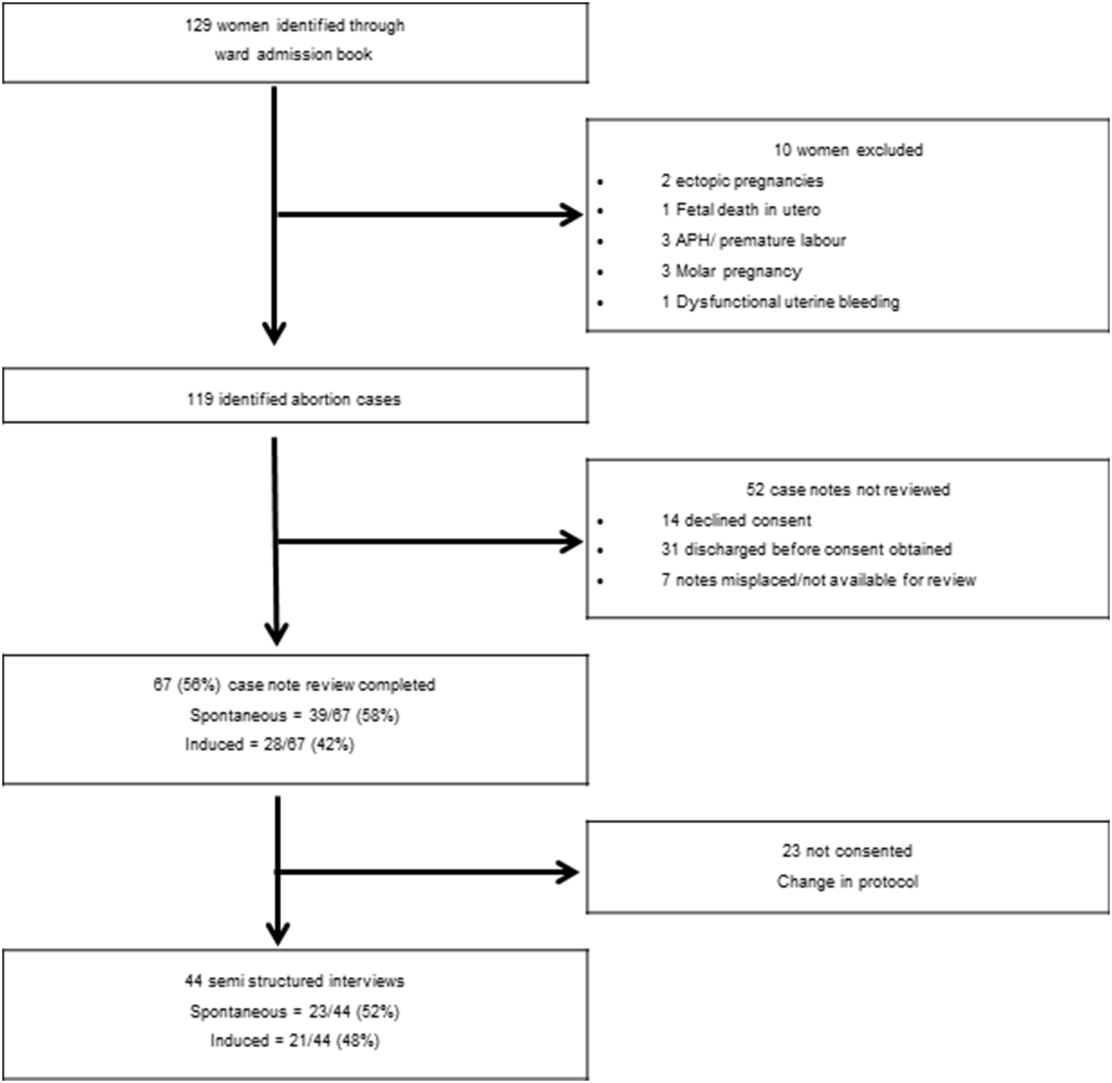
Identification of women admitted following spontaneous and induced abortion.

Over the six-month study period we identified 129 women who met the inclusion criteria. All women were identified through the ward admission book at the obstetric and gynaecology ward. Of these 129 women, 119 (92%) were identified as presenting with complications following a self-reported spontaneous or induced abortion (Figure 1). At least 24% (28/119) of all abortion related admissions were due to induced abortion.

### Semi structured interviews

Semi structured interviews were undertaken with 44 women out of a possible 79 recruited after the amendment to protocol, therefore the proportion of those consenting to participate in the semi structured interviews was 57% (44/79). Of these 44 semi structured interviews, 23 (52%) were conducted among women reporting spontaneous abortion and 21 (48%) were among women reporting induced abortion (Figure 1).

As a result of conducting semi structured interviews five women not reporting any interference with their pregnancy during the hospital admission process disclosed to the research midwife that they had interfered with their pregnancy. Of these five women, three were suspected as induced based on clinical examination; the remaining two had no report of clinical evidence that an induced abortion had taken place. Because of their disclosure during the semi structured interview they are reported here as induced abortion. One woman, who disclosed to the doctor at the time of admission that she had induced her abortion, reported a spontaneous abortion during her semi-structured interview. Based on the clinical findings and her disclosure at admission, she remains classified for this study as an induced abortion.

### Case note review

Case note review was undertaken for 56% (67/119) of all women identified. Case note review could not be undertaken for the remaining 46% (52/119) of women as consent was not obtained; 60% (31/52) were discharged home within 24 hours of admission (Figure 1). Of the 67 case notes reviewed, 39 (58%) women were identified as, or reported, a spontaneous abortion and 28 (42%) were identified as, or reported, an induced abortion (Figure 1). Seven women reporting induced abortion had their case notes reviewed before the introduction of the semi structured interviews.

#### Socio-demographic characteristics

Social and demographic details for all 67 women are shown in table 1. More than half of the women (35/67; 52%) were aged 15–24 years and were residing in Goroka district at the time of admission (35/67; 52%). Nearly three quarters (49/67; 73%) reported to be married. More than half of the women (41/67; 61%) were educated beyond grade seven and 15% (10/67) had received a tertiary or university level education; 25% (17/67) of women were a student (at either secondary school or university) at the time of admission. Compared to women who reported spontaneous abortion, those who had experienced induced abortion were significantly more likely to be aged 15–24 years (71% vs 39%, p = 0.0078); to be single (39% vs 3%, p = 0.0001); and were a student at time of admission to hospital (43% vs 13%, p = 0.0053).

**Table 1:** Social and demographic details : Case Note Review (n = 67).

		All women n = 67	Induced n = 28	Spontaneous n = 39	p-value
**Age**	15–24	35 (52%)	20 (71%)	15 (38%)	p = 0.0078
	25–34	26 (39%)	7 (25%)	19 (49%)	p = >0.05
	≥35	6 (9%)	1 (4%)	5 (13%)	p = >0.05
**Marital status**	Married	49 (73%)	13 (46%)	36 (92%)	p = >0.05
	Single	12 (18%)	11 (39%)	1 (3%)	p = 0.0001
	Separated	6 (9%)	4 (14%)	2 (5%)	p = >0.05
**Province of birth**	Eastern Highlands Province	51 (76%)	21 (75%)	30 (77%)	p = >0.05
	Other	15 (22%)	6 (21%)	9 (23%)	p = >0.05
	Not Known	1 (2%)	1 (4%)	0 (0%)	p = >0.05
**Current address**	Goroka district	35 (52%)	18 (64%)	17 (44%)	p = >0.05
	Other districts in Eastern Highlands	26 (39%)	9 (32%)	17 (44%)	p = >0.05
	Districts outside Eastern Highlands	6 (9%)	1 (4%)	5 (13%)	p = >0.05
**Education level**	University/tertiary	10 (15%)	4 (14%)	6 (15%)	p = >0.05
	Grade 11–12	6 (9%)	6 (21%)	0 (0%)	p = >0.05
	Grade 7–10	25 (32%)	8 (29%)	17 (44%)	p = >0.05
	Grade 4–6	12 (18%)	6 (21%)	6 (15%)	p = >0.05
	Grade 1–3	9 (13%)	3 (11%)	6 (15%)	p = >0.05
	No education	5 (7%)	1 (4%)	4 (10%)	p = >0.05
**Employment**	No paid job	4 (6%)	0 (0%)	4 (10%)	p = >0.05
	Subsistence farmer	26 (39%)	10 (36%)	16 (41%)	p = >0.05
	Housewife	10 (15%)	3 (11%)	7 (18%)	p = >0.05
	Teachers	2 (3%)	0 (0%)	2 (5%)	p = >0.05
	Student	17 (25%)	12 (43%)	5 (13%)	p = 0.0053
	Other paid work	8 (12%)	3 (11%)	5 (13%)	p = >0.05

#### Pregnancy history

Just over one third of women (25/67; 37%) had never given birth before; 27% (18/67) had given birth once in the past. More than half of the women (35/67; 52%) reported to be in their second trimester at the time of the abortion. Almost half of the women (32/67; 48%) reported that the pregnancy had been planned; 28% (19/67) said that the pregnancy was unintended and not wanted (Table 2).

**Table 2:** Obstetric History.

		All women	Induced	Spontaneous	p-value
		n = 67	n = 28	n = 39	
**Parity**	Nulliparous	25 (37%)	13 (46%)	12 (31%)	p = >0.05
	Para 1	18 (27%)	8 (29%)	10 (26%)	p = >0.05
	Para 2	9 (13%)	3 (11%)	6 (15%)	p = >0.05
	Para 3	9 (13%)	2 (7%)	7 (18%)	p = >0.05
	Para 4	3 (5%)	1 (4%)	2 (5%)	p = >0.05
	Para 5	3 (5%)	1 (4%)	2 (5%)	p = >0.05
**Current pregnancy**	Unplanned/unwanted	19 (28%)	18 (64%)	1 (3%)	p = <0.001
	Planned/wanted	32 (48%)	3 (11%)	29 (74%)	p = >0.05
	Unplanned/wanted	16 (24%)	7 (25%)	9 (23%)	p = >0.05
**Reported gestation at admission**	4–12 weeks	32 (48%)	11 (39%)	21 (54%)	p = >0.05
	13–20 weeks	32 (48%)	15 (54%)	17 (44%)	p = >0.05
	21–24 weeks	3 (4%)	2 (7%)	1 (3%)	p = >0.05

Parity and gestation at presentation to hospital was not significantly different between women reporting spontaneous abortion and those with induced abortion. Compared to women who reported spontaneous abortion, those who had experienced induced abortion were significantly more likely to report that the pregnancy was a mistake and the baby unwanted (64% vs 3%; p<0.0001).

#### Clinical presentation

Duration of symptoms before presentation varied between presenting the same day as onset of symptoms, up to four weeks. Most women (47/67; 70%) presented between 0–5 days (Table 3). Vaginal bleeding with associated abdominal pain was the most widely reported presenting complaint, reported by 61% (41/67) of all women.

**Table 3:** Clinical presentation.

		All women	Induced	Spontaneous	p-value
		n = 67	n = 28	n = 39	
**Duration of symptoms before presenting**	0–5 days	47 (70%)	17 (61%)	30 (77%)	p = >0.05
	6–10 days	11 (16%)	7 (25%)	4 (10%)	p = >0.05
	2–4 weeks	8 (12%)	4 (14%)	4 (10%)	p = >0.05
	Not known	1 (1%)	0 (0%)	1 (3%)	p = <0.001
**Pale complexion**	Any pale complexion	38 (57%)	15 (54%)	23 (59%)	p = >0.05
	Severe	5 (13%)	4 (27%)	1 (4%)	p = >0.05
	Moderate	20 (53%)	7 (47%)	13 (57%)	p = >0.05
	Mild	11 (29%)	4 (26%)	7 (30%)	p = >0.05
	Not specified	2 (5%)	0 (0%)	2 (9%)	p = >0.05
**Presenting complaint**	Just PV bleeding	10 (15%)	3 (11%)	7 (18%)	p = >0.05
	PV bleeding, abdominal pain	41 (61%)	13 (46%)	28 (72%)	p = >0.05
	PV bleeding, abdominal pain, fever	12 (5%)	10 (36%)	2 (5%)	p = 0.0013
	Other	4 (6%)	2 (7%)	2 (5%)	p = >0.05
**Signs of septic abortion**		19 (28%)	11 (39%)	8 (21%)	p = >0.05

There was no significant difference between onset of symptoms and presentation between the two groups of women. Compared to women who reported spontaneous abortion, those who had experienced induced abortion were significantly more likely to present with bleeding, pain and fever (36% vs 5%; p = 0.0013). Women presenting following induced abortion were significantly more likely to present with signs of severe anaemia (27% vs 4%; P = <0.001). There was no significant difference between the two groups in terms of clinical signs of septic abortion (Table 3).

#### Clinical management

97% (65/67) of women underwent a surgical procedure of whom 97% (63/65) had dilatation and curettage for retained products; two women had an exploratory laparotomy. Of the two who underwent laparotomy, one had inserted a stick into the vagina to induce an abortion three weeks earlier; she was found to have a pelvic abscess at laparotomy (Table 4). The other woman had a history of bleeding for two weeks prior to presentation and despite evidence of inflamed cervix and drainage of a pelvic abscess was identified as a spontaneous abortion.

**Table 4:** Duration of hospital stay and management.

	All women	Induced	Spontaneous	p-value
	n = 67	n = 28	n = 39	
**Duration of hospital stay**				
1–2 days	4 (6%)	1 (3.5%)	3 (8%)	P = >0.05
3–4 days	41 (61%)	18 (64%)	23 (59%)	P = >0.05
5–6 days	16 (24%)	6 (21%)	10 (26%)	P = >0.05
7–10 days	2 (3%)	1 (3.5%)	1 (2%)	P = >0.05
>10 days*	4 (6%)	2 (7%)	2 (5%)	P = >0.05
**Management**				
Dilatation and curretage	63 (94%)	26 (93%)	37 (95%)	P = >0.05
Exploratory laparotomy & drainageof pelvic abscess	2 (3%)	1 (3.5%)	1 (2.5%)	P = >0.05
Misoprostol	1 (1.5%)	1 (3.5%)	0	P = >0.05
Nil treatment	1 (1.5%)	0	1 (2.5%)	P = >0.05
**Received blood or blood products**	41 (61%)	20 (71%)	21 (54%)	P = >0.05

*2 women reporting spontaneous abortion stayed for 12 days; 2 women reporting induced abortion stayed for 20 & 21 days.

The majority of procedures were conducted by the obstetric registrar (53/66; 80%) or the senior obstetrician (11/66; 17%); the resident medical officer under took two procedures (Table 5). One woman received misoprostol for incomplete abortion and one woman required no intervention due to a complete abortion. Most women (64/67; 96%) received intravenous fluids, 61% (41/67) received blood or blood products. Those receiving blood or blood products were similar for both groups of women; 20 (49%) and 21 (51%) women respectively for induced and spontaneous cases.

**Table 5:** Doctor providing management/treatment (NB Only 66/67 women had an intervention).

	All proceduresn = 66	Dilatation & Curretagen = 63	Laparotomyn = 2	Misoprostoln = 1
Consultant obstetrician	11 (17%)	10 (16%)	1	0
Obstetric registrar	53 (80%)	51 (81%)	1	1
Resident Medical officer	2 (3%)	2 (3%)	0	0

Most women (41/67; 61%) stayed in hospital between for 3–4 days (Table 5). There were no significant differences relating to duration of hospital stay between the two groups of women.

Sixty three (94%) women received a method of family planning prior to discharge home of whom 62 received depo-provera; one opted for tubal ligation. Two women received family planning advice, one was referred onto a medical ward; family planning outcome was unknown for one woman.

There were no safe abortions undertaken at the hospital and no abortion related deaths recorded at the hospital during the six month study period.

### Unsafe, induced abortions

Of the 119 women identified, 35 (29%) were identified through the ward admission book as having had an induced abortion. However, data is only presented for the 28 women consenting to case note review. Semi structured interview was undertaken with three quarters of these women (21/28; 75%). Most women (21/28; 75%) reported an induced abortion at the time of admission. Five women (5/28; 18%) had clinical signs that an induced abortion had taken place, although this was not disclosed at the time of admission; two of these women did disclose interference with the pregnancy during the semi structured interview. Two women (2/28; 7%) who disclosed during their semi structured interview that they had induced their abortion had no clinical signs that the abortion had been induced.

Of all 28 induced cases identified, gestation at time of induction ranged between 7–24 weeks. More than half (17/28; 61%) took place in the second trimester, between 16–24 weeks gestation (Table 6).

**Table 6:** Gestation and reported method of abortion.

	All induced abortions n = 28	Gestation at abortion 7–12 weeks n = 11	Gestation at abortion 16–24 weeks n = 17
**Misoprostol**	14 (50%)	8 (73%)	6 (35%)
* PV misoprostol*	*5 (36%)*	*2 (25%)*	*3 (50%)*
* Oral misoprostol*	*2 (14%)*	*2 (25%)*	*-*
* PV & oral misoprostol*	*4 (28%)*	*2 (25%)*	*2 (33%)*
* Misoprostol, route unknown*	*3 (21%)*	*2 (25%)*	*1 (17%)*
**Physical means**	6 (22%)	1 (9%)	5 (29%)
**Traditional herbs**	3 (11%)	1 (9%)	2 (12%)
**Cultural beliefs/sorcery**	2 (7%)	-	2 (12%)
**Insertion of stick vaginally**	1 (3%)	1 (9%)	-
**Received injection from health care** **worker**	1 (3%)	-	1 (6%)
**Coffee**	1 (3%)	-	1 (6%)

Misoprostol was the most frequently reported method used to end pregnancy; 39% (11/28) of women reported using misoprostol. Both vaginal and oral routes of administration were mentioned with between two and five tablets taken (Table 6). Most women reported obtaining the misoprostol through a friend or relative working at a pharmacy; no women reported purchasing the misoprostol directly over the counter. Two women acquired the misoprostol through health care workers at hospitals outside of Goroka; another woman reported buying the misoprostol with a prescription which she had acquired through a health care worker at the hospital.

Six women (6/28; 21%) used physical means to end the pregnancy, including squeezing or tying a rope around the abdomen, excessive exercise, falling onto the abdomen, hard physical work and insertion of a stick into the vagina. The use of traditional herbs, a concoction frequently consisting of herbs, leaves and ginger which is boiled up and drunk resulting in vomiting and abdominal cramps was reported by three women (3/28; 11%). Two women reported that interference with the pregnancy had taken place through the use of witchcraft and evil spirits from another family member towards her (some areas of PNG have very strong cultural and spiritual belief relating to witchcraft, sorcery and evil spirits, especially in relation to understanding health and well-being). One woman reported receiving an injection from a health care worker at an undisclosed hospital. While she did not know what the injection was she stated that it was not a family planning injection. One woman mentioned that there had been some interference with the pregnancy due to physical violence from her husband and one described how she had drunk copious amount of strong instant coffee, bringing on vomiting and abdominal cramps (Table 6). None of the women reported trying or using more than one abortion method.

## Discussion

At least 24% (28/119) of all abortion cases identified during the study period were due to induced abortion. Those identified as induced abortion were significantly more likely to be younger, single, in education at the time of the abortion, and reported that the pregnancy had been a mistake and was not wanted, compared to women reporting spontaneous abortion. Women presenting following an induced abortion were significantly more likely to present with abdominal pain, vaginal bleeding and fever, compared to those reporting a spontaneous abortion. They were also significantly more likely to be anaemic. Clinical management and outcome was similar for women in both groups with no significant differences in their management or duration of hospital stay.

Misoprostol to end an unwanted pregnancy is becoming increasingly used in both developed and developing countries [4], [8] and has been previously reported in the setting for this study [27]. Table 7 outlines correct regimes for the use of misoprostol-only induced abortion. Although misoprostol used alone is a safe means of medically induced abortion, it is most often safe to do so when factors of gestation and correct dose are followed [3], [28]. In the absence of adequate supervision from a skilled health worker the situation becomes riskier [3], [28]; and the use of sub optimal misoprostol regimes may result in several days of hospitalisation [29]. Of the 14 women identified in our study who used misoprostol, 12 followed incorrect regimes and six were in their second trimester of pregnancy. The only two women in our study who received a dose of misoprostol appropriate for their gestation received the misoprostol from hospital based health care workers. Both of these women were in their second trimester. Most of the women in our study who used misoprostol to induce their abortion were at risk of complications due to incorrect use of misoprostol, and second trimester abortion. What is unknown in this setting is whether women are accessing misoprostol earlier in their pregnancy, and using it correctly and more safely to end a first trimester abortion, therefore having no need to present for hospital level care.

**Table 7:** Recommended guidelines for Misoprostol-only medical abortion [Source: WHO 2012].

Gestation	Recommended method
***Pregnancies up to 12 weeks***	800 mcg of misoprostol administered vaginally or sublingually.
***(84 days)***	Repeat at 3 hourly intervals, but for no longer than 12 hours.
***Pregnancies of gestational age over*** ***12 weeks (84 days)***	400 mcg of misoprostol administered vaginally or sublingually.
	Repeat at 3 hours for up to five doses.
**Pregnancies beyond 24 weeks**	No specific dosing recommendations due to lack of clinical studies.
	But dose of misoprostol should be reduced, due to the greater sensitivity of the uterus to prostaglandins.

Clinical management varied very little between the two groups of women and there was no different clinical management for managing induced abortion- whether women had used misoprostol or other means to end the pregnancy. National guidelines for incomplete abortion in PNG specify that incomplete abortion should be treated with either manual vacuum aspiration (MVA) or misoprostol [30]. Since its addition to the WHO essential drug list in 2011, misoprostol is available in all hospitals in PNG for use in managing incomplete abortions, induction of labour and prevention and treatment of postpartum haemorrhage. In this study only one woman was treated for incomplete abortion with misoprostol. The training and use of MVA techniques is still underway for many obstetricians in PNG, however dilatation and curettage is widely practiced, as identified in this study. Dilation and curettage requires trained, skilled health personnel, management that may consequently place an additional burden on a struggling workforce. MVA, when undertaken in the first trimester, is quicker and associated with less blood loss when compared to dilatation and curettage [31]. In addition, it may be carried out successfully by specifically trained nurses and midwives [32], [33], thus reducing the clinical workload for doctors. Given the safety and effectiveness of both MVA and misoprostol in the first trimester [34], [35] it is possible that some women presenting for post abortion care could be managed without the need of senior medical staff.

While grounds on which abortion can be legally performed has broadened in many developing countries, in countries where it remains illegal abortions frequently continue to take place in unsafe circumstances [36]. In many settings, the shift in legislative frameworks allowing for more legal abortions has resulted in a decline in the overall abortion rate, globally, however there has been little decline in unsafe abortions [37]. As with many developing countries, access to safe abortion in PNG is restricted due to the legal framework and lack of availability of safe abortion services through the government system. While a safe abortion should be available if a woman’s physical or mental health is at risk, in reality safe abortions rarely take place, through government or non-government health services [17]. Safe abortions available through private clinics are restricted to the few practitioners in the urban settings offering the service at a cost unaffordable to most Papua New Guineans. As such, access to safe abortion in PNG remains unavailable and inaccessible to the vast majority, due not only to socio-cultural, geographical and financial constraints but also a lack of understanding relating to the legality of abortion, many women, and health care workers in PNG believe abortion to be “illegal” [26]. This situation is similar to other countries with restrictive abortion laws and availability of safe abortions almost exclusively restricted to those who can afford it [38]. Many of the factors inhibiting access to safe abortion services also play a role in women accessing health services for post abortion care, with many afraid of repercussions from health staff and the legal framework surrounding unsafe abortion practices [5], [26], [37].

Global estimates indicate that for every woman presenting to a health facility for management of complications following an unsafe abortion, several other women do not reach a health facility for post abortion care services [1]. Given the poor uptake of maternal health services generally in PNG, for example the supervised birth rate is 53% and the contraceptive prevalence rate is 24% [18], it is reasonable to assume that our study only highlights a small proportion of women undertaking an unsafe abortion, with many more women never reaching a health facility and therefore never part of any official statistics. Given the wide use of misoprostol among our study participants it is a possibility that some women accessed misoprostol resulting in a safe, albeit illegal abortion and therefore not requiring hospital level care. There is also the possibility that some unsafe abortions result in the death of the woman, perhaps never being recognised as a maternal mortality or reported as an abortion related death. There were no abortion-related maternal deaths recorded at the hospital during the study period. While abortion related maternal deaths have previously been reported from the same study setting, the ten deaths, from a total of 29 recorded over a three year and five month period were recorded as deaths relating to haemorrhage and abortion-related sepsis; only three were confirmed as abortion related [17].

Despite its limitations, this is the first study from PNG that has attempted to capture both spontaneous and induced abortions presenting for hospital level care, highlighting the clinical presentations and management of a vulnerable group of women. The data presented is from one hospital setting undertaken over a relatively short period of time. Despite our best efforts we were only able to review just over half of the case notes for women admitted following an abortion. Being unable to obtain informed consent was due to a number of reasons, including restricted access to the ward, doctors ward rounds and theatre lists and women being discharged home within a short time frame. The early discharge home of some of the women may indicate that these particular women may have presented with less complicated morbidity; some may have presented earlier following onset of symptoms or may have been in the earlier stages of pregnancy. The use of semi structured interviews was included during the study as we felt we were missing some of the induced cases. Following this amendment to study design we identified just an additional two cases of induced abortion that would otherwise have been missed. Most women presenting following an induced abortion did report as such at the time of admission. Others were identified based on their history and clinical examination. However, it is possible that we missed other women presenting following induced abortion due to the limited number of case notes reviewed. This study was limited by the amount of data we were able to collect and we recognise that a larger study and sample size would have allowed us to more fully develop the findings and describe patterns and differences between the two groups of women. As this was a hospital based study, we captured only women seeking specialist medical care.

Aside from the limitations this study highlights the burden on the hospital and hospital staff for clinical management of all women presenting following both spontaneous and induced abortion. It is hoped that the findings will help assist with policy and planning, particularly in relation to management of incomplete abortion and to reproductive health services for women.

## Conclusion

Our study only highlights the plight of women who present to hospital for post abortion care. As in many developing countries women in PNG are vulnerable to unplanned pregnancies and subsequently unsafe abortions. In a country with a low CPR and high unmet need for family planning, women of all ages need access to contraceptive information and services to avoid, postpone or space pregnancies. In the absence of this, women are resorting to unsafe means to end an unwanted pregnancy. In view of the proportion of women admitted to hospital following unsafe abortion, women need access to safe, effective means of abortion. In addition, the training of clinical staff in the use of MVA and misoprostol for incomplete abortions could reduce the burden on an already constrained health system. Knowledge of the legal framework on induced abortion in PNG needs to be improved in the community and among medical staff to improve access for those women eligible for a legal induced abortion. Improved access to safe abortion services together with the review of post abortion care services in PNG could help in reducing the burden of maternal mortality and morbidity from unsafe, induced abortions.

## Supporting Information

Figure S1 **Semi structured interviews with women.**
(TIF)
